# 

**Published:** 1898-10

**Authors:** 


### CONTENTS.

ORIGINAL COMMUNICATIONS. PAGE
Anæsthetics. By H. H. Burchard, M.D., D.D.S., Philadelphia . . . . . . . . 629
Double Resection of the Lower Maxilla. By Eugene S. Talbot, M.D., D.D.S. . 640
Associate Lesions of the First Dentition. By Harry L. Belcher, D.D.S., Buffalo, N. Y. . . . . . . . . . . . . . . . . . . . . . . . . . . 641
Reminiscences of a European Trip to the International Medical Congress at Moscow, Russia, August 19 to 26, 1897, as a Delegate from the American Medical Association and the Odontological Society of Pennsylvania. By W. G. A. Bonwill, D.D.S., Philadelphia . . . . . . . . . . . . . . . . . . 646
Patents *versus* No Patents. By Dr. G. Alden Mills, New York . . . . . . . 667

ABSTRACTS AND TRANSLATIONS.
A New Factor in Erosion. By Arthur S. Underwood, M.R.C.S., L.D.S. . . . 669
A Series of Four Cases of swallowing Artificial Teeth treated in the Royal Southern Hospital, Liverpool, during the last Six Months. By John Owen, M.B. . . . 671
New Theory in regard to Caries of the Teeth . . . . . . . . . . . . . . . . . 673
General Medical Council, Great Britain—Abstract of Discussion upon Materia Medica . . . . . . . . . . . . . . . . . . . . . . . . . . . . . . 675

REPORTS OF SOCIETY MEETINGS.
Academy of Stomatology . . . . . . . . . . . . . . . . . . . . . . . . . . 678

EDITORIAL.

The Recent Conventions . . . . . . . . . . . . . . . . . . . . . . . . . . 687

General Medical Council of Great Britain . . . . . . . . . . . . . . . . . 690

BIBLIOGRAPHY . . . . . . . . . . . . . . . . . . . . . . . . . . . . . . . 691

NOTES AND COMMENTS . . . . . . . . . . . . . . . . . . . . . . . . . . . 693

CURRENT NEWS.
National Association of Dental Examiners . . . . . . . . . . . . . . . . . 696
Northeastern Dental Association . . . . . . . . . . . . . . . . . . . . . 696

LIBRARY

SURGEON GENERAL'S OFFICE

